# The complete chloroplast genome and phylogenetic status of *carpesium nepalense* less. 1831 (asteraceae)

**DOI:** 10.1080/23802359.2025.2528575

**Published:** 2025-07-07

**Authors:** Yu Su, Jingyi Peng, Yining Di, Hancaiyuan Zheng, Xianhan Huang, Lufeng Liu

**Affiliations:** ^a^ College of Resources and Environment, Yunnan Agricultural University, Kunming, Yunnan, China; ^b^ State Key Laboratory for Conservation and Utilization of Bio-Resource in Yunnan, Kunming, Yunnan, China; ^c^ CAS Key Laboratory for Plant Diversity and Biogeography of East Asia, Kunming Institute of Botany, Chinese Academy of Sciences, Kunming, China

**Keywords:** Carpesium, medicinal value, chloroplast genome, phylogenetic analysis

## Abstract

The complete chloroplast genome of *Carpesium nepalense* (Asteraceae), an important medicinal herb, was characterized. It exhibits a typical quadripartite structure with inverted repeats (24,947 bp), a large single-copy region (82,870 bp), and a small single-copy region (18,473 bp), with 37.7% GC content. The genome contains 133 genes, including 88 protein-coding, 37 tRNA, and 8 rRNA genes. Phylogenetic analysis confirms *Carpesium* as monophyletic, with *C. nepalense* genetically closest to *C. cernuum, C. faberi, C. lipskyi*, and *C. longifolium*. This work enriches the molecular database of *Carpesium* and provides new genomic resources for the development and utilization of medicinal herbs..

## Introduction

*Carpesium* L. (1753) is a herbaceous plant in the family Asteraceae, comprising approximately 20 species worldwide, primarily distributed across Eurasia. China represents the center of diversification of the genus, with 16 species recorded, six of which are endemic (Chen and Anderberg 2011). Among them, *C. nepalense* Less. (1831) has been reported to have antimicrobial and anticancer activities, and compounds isolated from this species have also shown potential in treating Alzheimer’s disease (Burki et al. 2024); some other species (such as *C. abrotanoides* L. 1753 and *C. cernuum* L. 1753) contain sesquiterpenoids, which are important as folk medicines in the treatment of folliculitis, toothache, and fever (Yang 2016; Zhang et al. 2015).

The chloroplast genome of land plants shows a highly conserved structure and gene organization, consisting of a single circular molecule with a quadripartite structure. It usually includes a pair of inverted repeat regions (IRs), a large single-copy (LSC) region, and a small single-copy (SSC) region (Daniell et al. 2016). Chloroplast genomes have served as effective molecular resources for improving species identification and have developed into ideal tools for resolving phylogenetic relationships at different taxonomic levels in plants (Jansen et al. 2007). Shi et al. (2022) performed a comparative genomic analysis of *Carpesium*, focusing on *C. abrotanoides*, *C. cernuum* and *C. faberi* C. Winkl. (1895), identifying five candidate DNA barcodes suitable for species discrimination. Subsequently, the chloroplast genomes of *C. lipskyi* C. Winkl. (1895) and *C. longifolium* F. H. Chen & C. M. Hu (1974) have also been reported by Li et al. (2024) and Chen et al. (2025), respectively. These studies have laid a solid foundation for exploring the genetic structure and diversity of chloroplast genomes within this genus. In this context, we continued our efforts to characterize the chloroplast genomes of *C. nepalense*, representing a further step toward enriching the genetic pool for *Carpesium*.

## Material and methods

### Plant sampling, DNA extraction and sequencing

**Figure 1.**
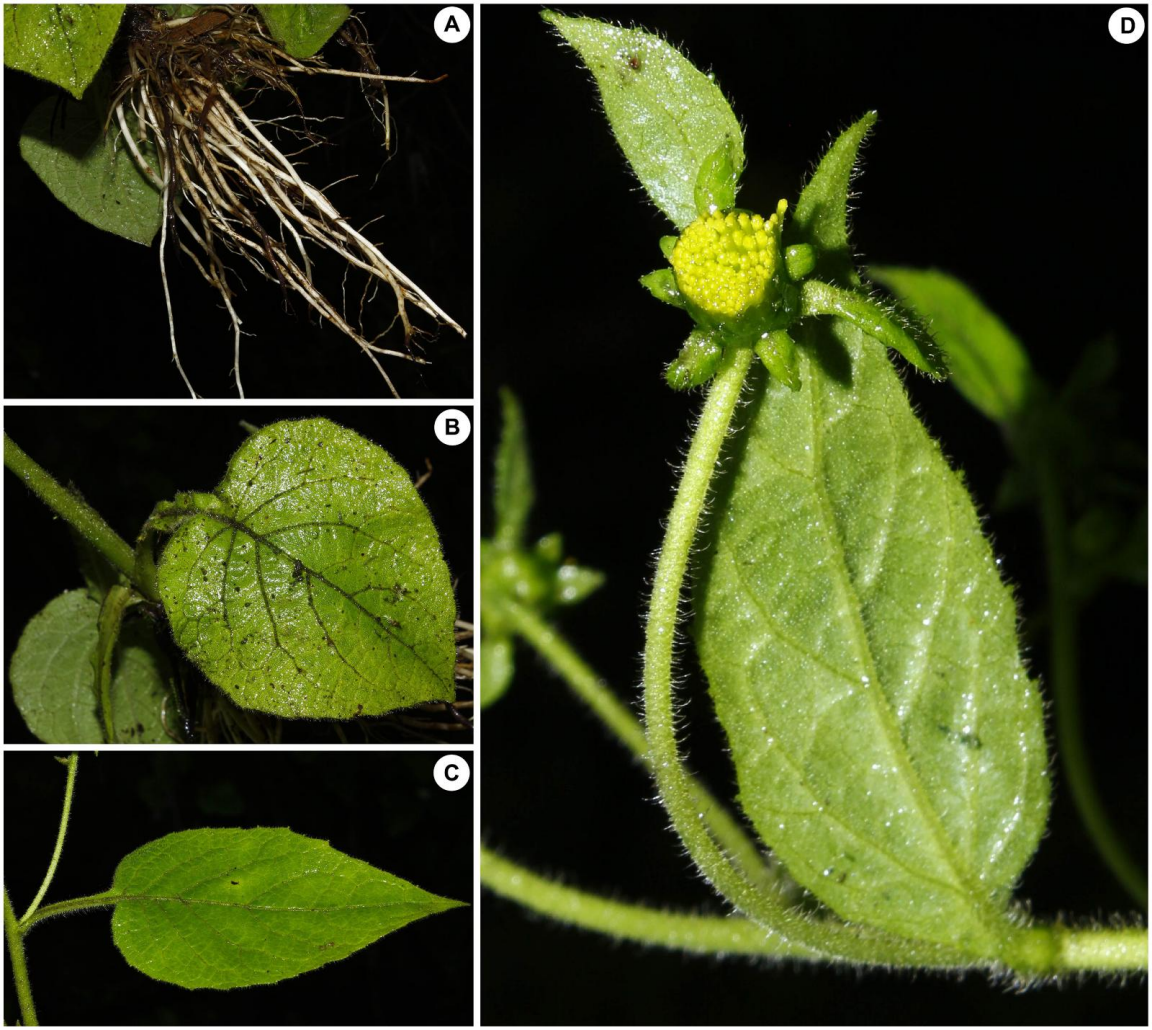
Photographs of *carpesium nepalense* taken during field investigation in Longzi County, Tibet, China (provided by liu qun). (A) Root. (B, C) Basal and cauline leaves. (D) Capitate flower.

*C. nepalense* specimens (Figure 1) were sampled from Longzi County, Tibet, China (93°06′12.92″E, 28°31′41.59″N), and its corresponding voucher specimens (YM-2-2, appraiser: Jingyi Peng) have been deposited in the Herbarium of Kunming Institute of Botany, Chinese Academy of Sciences (KUN) (contact: Tao Deng, dengtao@mail.kib.ac.cn). Fresh leaves collected during field investigations were used as the source material for this study. High-quality total DNA was extracted from silica-dried leaves at Novogene (Beijing, China). Sequencing libraries were constructed using the NEB Next^®^ Ultra DNA Library Prep Kit for Illumina^®^ (Ipswich, Massachusetts, USA) following the manufacturer’s recommendations. The prepared libraries were subsequently sequenced on the Illumina HiSeq 4000 platform.

### Assembly and annotation of the chloroplast genome

We assembled the chloroplast genome of *C. nepalense* from 1.36 Gb of clean data employing GetOrganelle v1.7.4.1 (Jin et al. 2018), with the following parameters: -F plant_cp -R 40 -t 10 -k 75,95,115,127. Among these parameters, ​​"F"​​ denotes the preconfigured reference chloroplast genome (plant_cp) for assembly, ​​"R"​​ represents the iteration rounds for extracting chloroplast sequencing reads, ​​"t"​​ indicates the number of CPUs employed for parallel computation, and ​​"k"​​ designates the k-mer size invoked during de novo genome assembly using SPAdes. The coverage depth was estimated by mapping readings onto the genome sequence using bowtie2 (Langmead and Salzberg 2012). The genome sequence was annotated on the Geseq online website (https://chlorobox.mpimp-golm.mpg.de/geseq.html) (Tillich et al. 2017). We manually checked the annotated genes in Geneious v.9.0.2, with special attention given to the verification of start and stop codons, using previously published chloroplast genomes of *Carpesium* by Shi et al. (2022), Li et al. (2024), and Chen et al. (2025) as references. Finally, the newly generated chloroplast genome of *C. nepalense* in this study was deposited in the NCBI GenBank database (https://www.ncbi.nlm.nih.gov) under accession number PV325052. Besides, we used Chloroplot (Zheng et al. 2020) and Chloroplast Genome Viewer (CPGView) (Liu et al. 2023) to draw the circular genome map and splicing genes, respectively.

### Phylogenetic analysis

We integrated the chloroplast genome of *C. nepalense* with 12 genome accessions of other related taxa from the NCBI based on the datasets reported in previous studies (Chen et al. 2025; Li et al. 2024; Shi et al. 2022), and *Anthriscus cerefolium* (L.) Hoffm (Li et al. 2024) was selected as the outgroup. All genome sequences were aligned using MAFFT (Katoh and Standley 2013) in Geneious v.9.0.2 with default parameters (Algorithm: Auto; Scoring matrix: 200PAM/*k* = 2; Gap open penalty: 1.53; Offset value: 0.123). Maximum likelihood (ML) analysis was conducted using IQ-TREE v.2.0.3 (Nguyen et al. 2015) to construct a ML phylogenetic tree, with the following command on a Linux platform: iqtree -s sequence alignment.fasta -m MFP -bb 1000.

## Result

**Figure 2.**
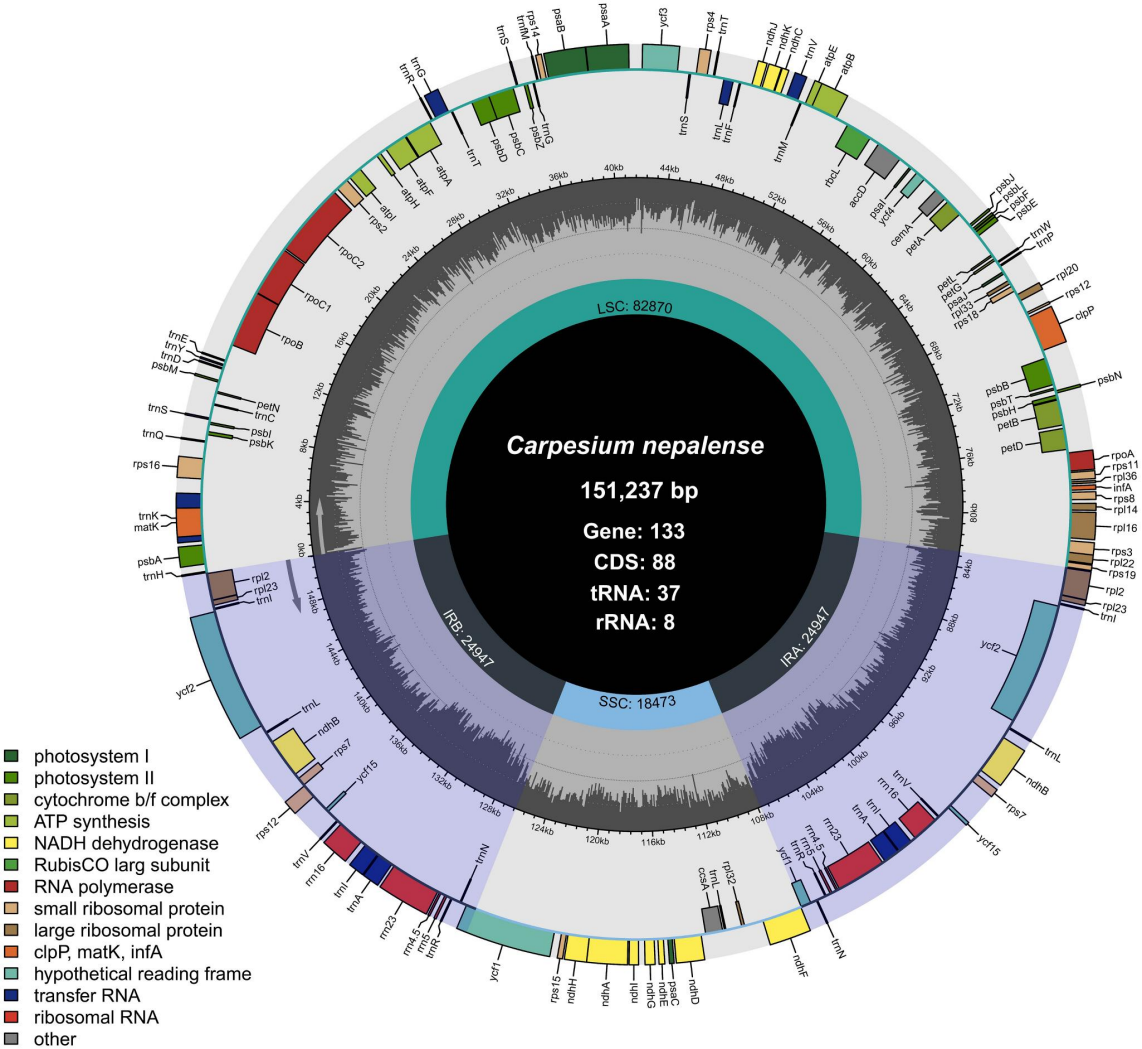
Circular chloroplast genome map of *carpesium nepalense* was drawn using chloroplot (Zheng et al. 2020) to show genes in each region (LSC, SSC and IRs). Transcription directions for the inner and outer genes are clockwise and anticlockwise, respectively, and each functional group of genes is distinctively color-marked. In the inner circle, the darker gray shades represent the GC content, and the lighter gray shades signify the AT content.

The newly generated complete circular chloroplast genome of *C. nepalense* exhibits a typical quadripartite structure with an average sequencing coverage depth of 190.92× (Figure S1). The genome is 151,237 bp in length and comprises a pair of 24,947 bp inverted repeat regions (IRs) separated by a large single-copy (LSC) region of 82,870 bp and a small single-copy (SSC) region of 18,473 bp (Figure 2). The overall GC content of the genome is 37.7%, while the IRs show a significantly higher GC content (43.1%) compared to the LSC (35.9%) and SSC (31.3%). After manual examination of the annotations, a total of 133 genes were identified, including 88 protein-coding genes (CDS), 37 tRNA genes, and 8 rRNA genes. Among these, 11 genes are cis-splicing, including *rps*16, *rpo*C1, *atp*F, *ycf*3, *clp*P, *pet*B, *pet*D, *rpl*16, *rpl*2, *ndh*B and *ndh*A, whereas *rps*12 is identified as a trans-splicing gene (Figure S2). In addition, *ycf*1 gene is the only one spanning the boundary between the IR and SC regions.

**Figure 3.**
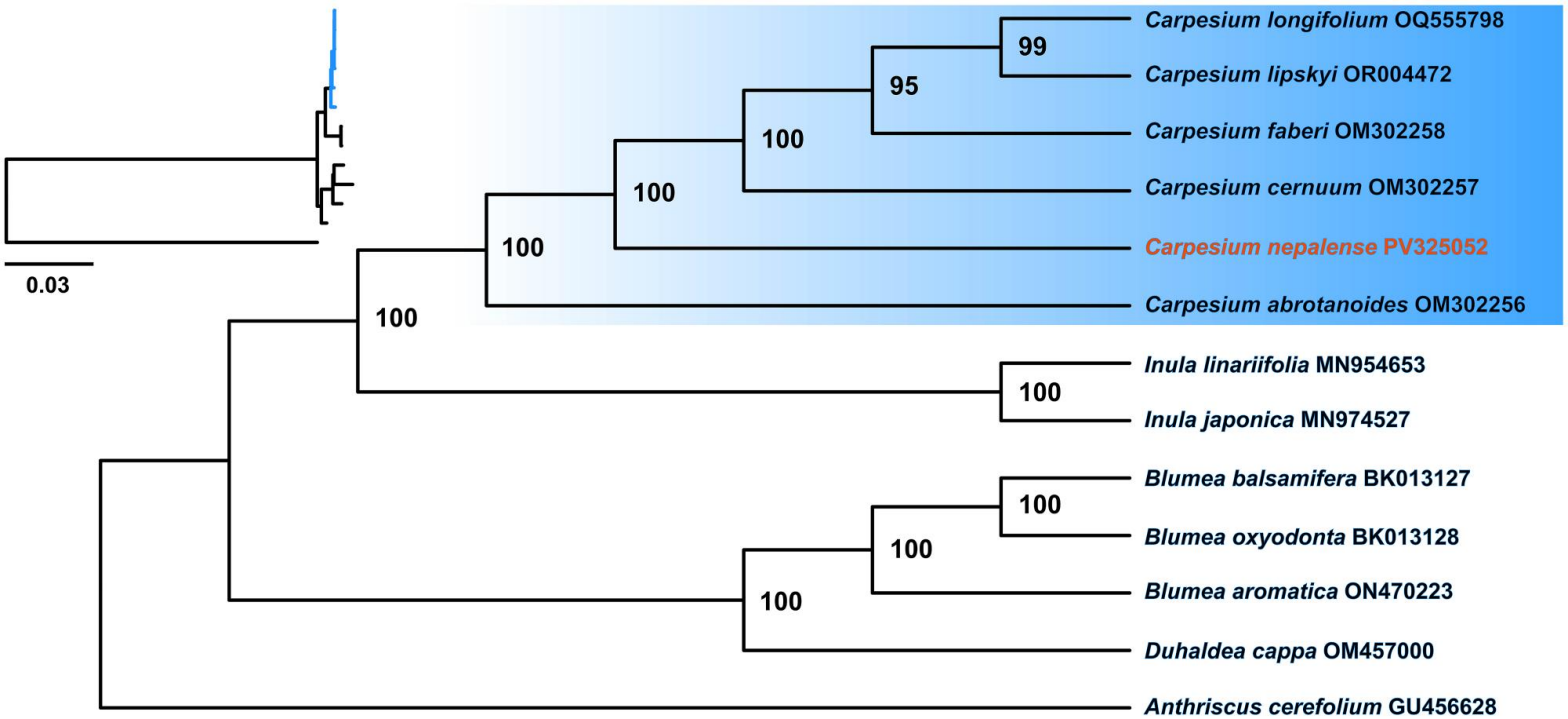
ML phylogenetic tree constructed using IQ-TREE v.2.0.3 (Nguyen et al. 2015) with 1000 bootstrap replicates under the best model of K3Pu + F + G4 selected by the bayesian information criterion (BIC), with a phylogram of the tree presented in the upper left corner. Bootstrap support (BS) values are showed at each node, and the phylogenetic position of *carpesium nepalense* is highlighted in red. Sources of chloroplast genomic data are as follows: *Anthriscus cerefolium* GU456628 (Li et al. 2024), *duhaldea cappa* OM457000 (Li et al. 2024), *blumea aromatica* ON470223 (Li et al. 2024), *blumea balsamifera* BK013127 (Abdullah et al. 2021), *blumea oxyodonta* BK013128 (Abdullah et al. 2021), *inula japonica* MN974527 (Li et al. 2024), *inula linariifolia* MN954653 (Li et al. 2024), *carpesium abrotanoides* OM302256 (Shi et al. 2022), *carpesium cernuum* OM302257 (Shi et al. 2022), *carpesium faberi* OM302258 (Shi et al. 2022), *carpesium longifolium* OQ555798 (Chen et al. 2025), and *carpesium lipskyi* OR004472 (Li et al. 2024).

Under the K3Pu + F+G4 optimal model selected by Bayesian information criterion (BIC), the ML phylogenetic tree was constructed with high bootstrap support (BS ≥ 95, Figure 3). Phylogenetic analysis indicated that all *Carpesium* species formed a monophyletic clade (BS = 100) and was sister to the *Inula* L. (BS = 100). Within the *Carpesium* lineage, *C. nepalense* established a sister relationship to *C. cernuum*, *C. faberi*, *C. lipskyi* and *C. longifolium* (BS = 100).

## Discussion and conclusions

The complete chloroplast genome of *C. nepalense* is comparable in genome size (151,237–151,389 bp) and gene content (127–133) to other *Carpesium* species (Chen et al. 2025; Li et al. 2024; Shi et al. 2022), reflecting the relatively conserved nature of chloroplast genomes within this genus. Previous molecular studies based on several DNA markers suggested that *Carpesium* is polyphyletic (Englund et al. 2009; Nylinder and Anderberg 2015), whereas complete chloroplast genome sequences with more informative loci have brought new insights into the phylogenetic study for *Carpesium*. Our phylogenetic analysis strongly supported the monophyly of *Carpesium*, and the topology was consistent with previous findings using chloroplast genomic data. However, according to classical taxonomy, *C. longifolium*, *C. abrotanoides* and *C. faberi* were traditionally assigned to Sect. *Abrotanoides*, while *C. cernuum*, *C. lipskyi* and *C. nepalense* belonged to Sect. *Carpesium*. Our results indicated that interspecific relationships within each section were not clearly resolved, seeming to deviate from the morphological taxonomic framework. Therefore, additional molecular data (e.g. single-copy nuclear genes, genomes, transcriptomes, etc.) are still needed to clarify the infrageneric relationships of *Carpesium* in order to further validate the rationality of the traditional taxonomy for dividing sections. In conclusion, this study successfully characterized the chloroplast genome of *C. nepalense*, providing valuable genomic resources for future study on the systematic evolution of *Carpesium*, as well as for the development and utilization of this species as a source of medicinal plants.

## Supplementary Material

Appendices.docx

## Data Availability

The genome sequence data that support the findings of this study are openly available in NCBI GenBank (https://www.ncbi.nlm.nih.gov/) under the GenBank accession PV325052. The associated BioProject, SRA and BioSample numbers are PRJNA1238255, SRR32772063 and SAMN47464588, respectively.
