# Supplementary material for: The complete chloroplast genome and phylogenetic status of carpesium nepalense less. 1831 (asteraceae)
Source: Mitochondrial DNA B Resour. 2025 Jul 7;10(8):663–7. doi: 10.1080/23802359.2025.2528575 (PMC12239104; doi:10.1080/23802359.2025.2528575)
Supplement: Appendices.docx [file TMDN_A_2528575_SM8541.docx]

**The complete chloroplast genome and phylogenetic status of *Carpesium nepalense* Less. 1831 (Asteraceae)**

Yu SU^1, 2, #^, Jingyi Peng^3, #^, Yining Di^1^, Hancaiyuan Zheng^1^, Xianhan Huang^3, *^, Lufeng Liu^1, 2, *^

1. College of Resources and Environment, Yunnan Agricultural University, Kunming, Yunnan, China
2. State Key Laboratory for Conservation and Utilization of Bio-Resource in Yunnan, Kunming, Yunnan, China
3. CAS Key Laboratory for Plant Diversity and Biogeography of East Asia, Kunming Institute of Botany, Chinese Academy of Sciences, Kunming, China

^#^Yu SU and Jingyi Peng contributed equally to this work.

**Correspondence:** Xianhan Huang, huangxianhan@mail.kib.ac.cn; Lufeng Liu, llf8839@sina.com

**This Appendix File Includes:**

Figure S1 to S2

**Content:**

**Figure S1.** The coverage depth figure of the *Carpesium nepalense* chloroplast genome. The horizontal coordinate is the base of the chloroplast genome, and the vertical coordinate is the depth of sequencing corresponding to that base.

**Figure S2.** The schematic map of the cis-splicing (*rps*16, *rpo*C1, *atp*F, *ycf*3, *clp*P, *pet*B, *pet*D, *rpl*16, *rpl*2, *ndh*B and *ndh*A) and the trans-splicing (rps12) genes in the *Carpesium nepalense* chloroplast genome.


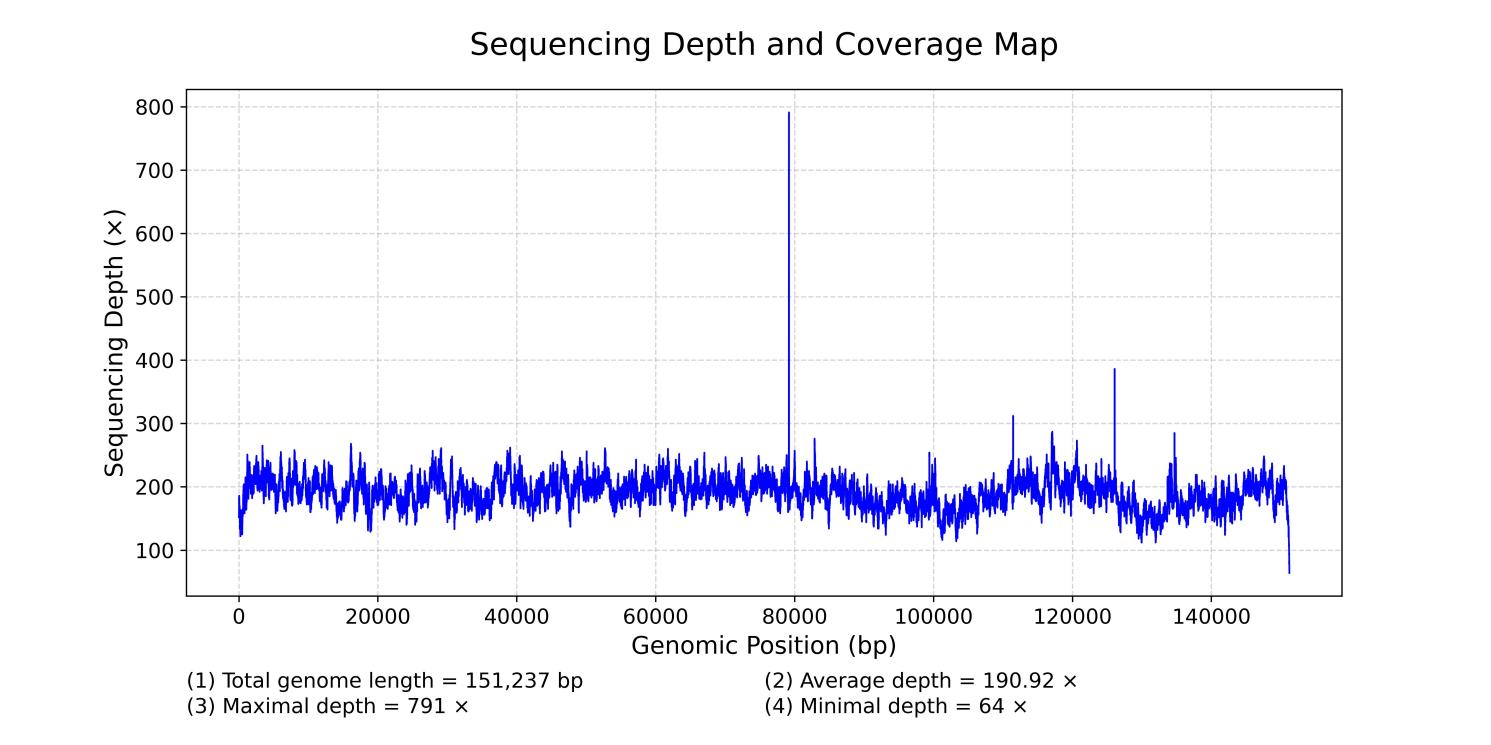


**Figure S1.** The coverage depth figure of the *Carpesium nepalense* chloroplast genome. The horizontal coordinate is the base of the chloroplast genome, and the vertical coordinate is the depth of sequencing corresponding to that base.


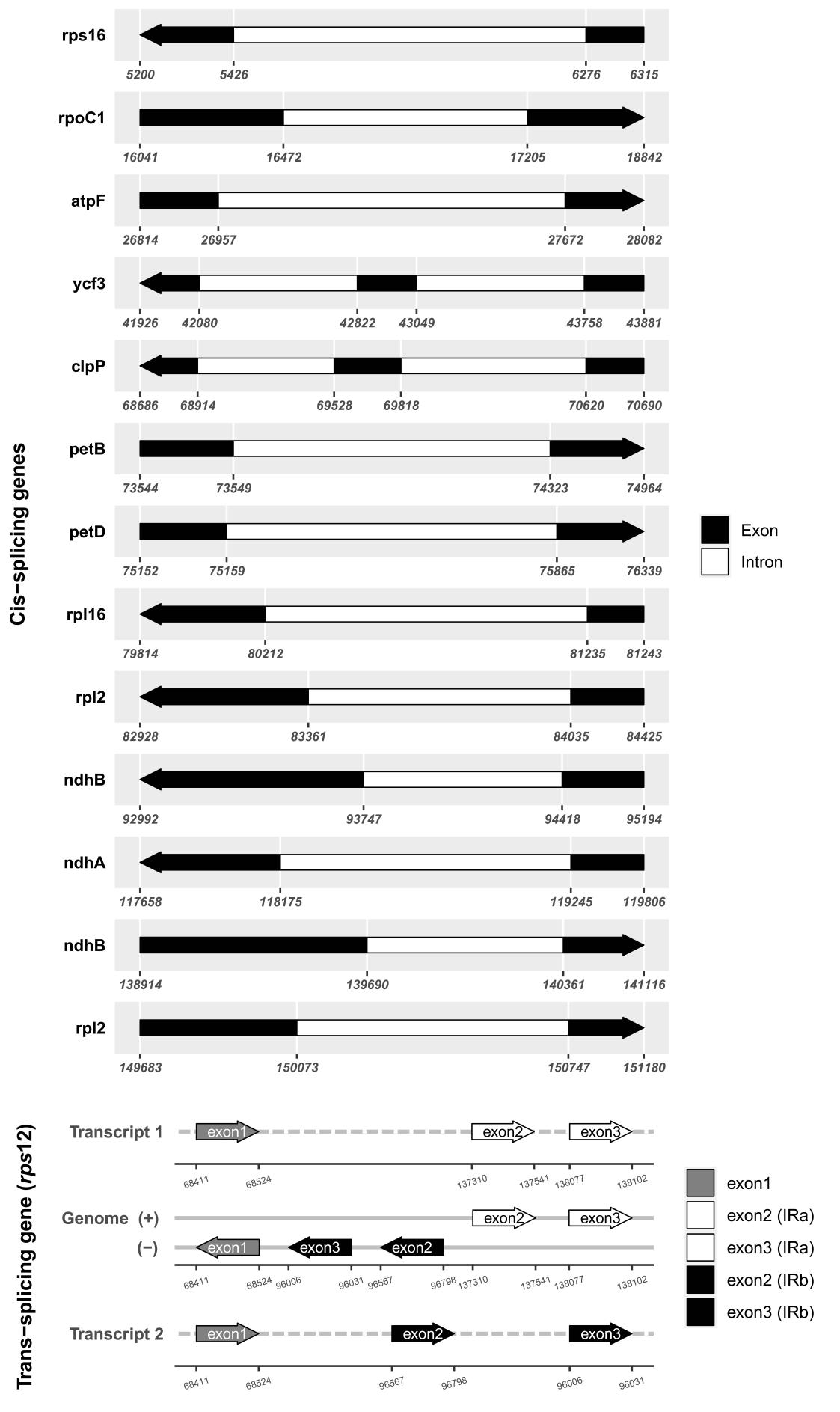


**Figure S2.** The schematic map of the cis-splicing (*rps*16, *rpo*C1, *atp*F, *ycf*3, *clp*P, *pet*B, *pet*D, *rpl*16, *rpl*2, *ndh*B and *ndh*A) and the trans-splicing (rps12) genes in the *Carpesium nepalense* chloroplast genome.
